# Crossing the finish line: a narrative inquiry into the role of exercise in patients with anorexia nervosa

**DOI:** 10.1186/2050-2974-2-S1-O3

**Published:** 2014-11-24

**Authors:** Sarah Young, Paul Rhodes, Stephen Touyz, Phillipa Hay

**Affiliations:** 1 University of Sydney, Sydney, Australia; 2 University of Western Sydney, Sydney, Australia

## 

The current study explored the role of exercise in the treatment and recovery process of Anorexia Nervosa (AN). 24 female participants completed the study: 10 women currently in treatment for AN; 7 partially recovered and 7 fully recovered, according to strict criteria. Participants undertook a structured interview assessing eating disorder psychopathology and a semi-structured interview where they were invited to share their story of their illness, with a focus on exercise. Narrative inquiry analyses revealed exercise can be a significant part of the individual’s life in various stages - premorbidly, during the illness, in treatment and recovery processes. Analyses demonstrated important themes including: rapid transformation into compulsive exercise during AN; importance of containment processes during treatment, appropriate limit setting and accountability in early stages of recovery; and the resumption of healthy exercise in full recovery. Results were developed into a model of exercise depicting these themes. Clinical implications to support re-integrating healthy exercise in treatment include the use of psycho-education and structured exercise interventions in treatment services. The findings emphasize the need for further clinical guidelines to ensure consistency in management of compulsive exercise in AN patients. Ongoing interpersonal and therapeutic support is required for patients to re-establish healthy exercise in recovery.

This abstract was presented in the **Peter Beumont Young Investigator award finalist** stream of the 2014 ANZAED Conference.

